# Do female *Nicrophorus vespilloides* reduce direct costs by choosing males that mate less frequently?

**DOI:** 10.1098/rsbl.2015.1064

**Published:** 2016-03

**Authors:** P. E. Hopwood, G. P. F. Mazué, M. J. Carter, M. L. Head, A. J. Moore, N. J. Royle

**Affiliations:** 1 Centre for Ecology and Conservation, College of Life and Environmental Sciences, University of Exeter, Cornwall Campus, Penryn TR10 9EZ, UK; 2 Department of Collective Behaviour, Max Planck Institute for Ornithology, University of Konstanz, Konstanz, Germany; 3 Department of Biology, University of Konstanz, Konstanz, Germany; 4 Centro Nacional del Medio Ambiente, Universidad de Chile, Avenida Larrain 9975, La Reina, Santiago, Chile; 5 Division of Evolution, Ecology and Genetics, Research School of Biology, Australian National University, Acton, Australian Capital Territory 0200, Australia; 6 Department of Genetics, University of Georgia, Athens, GA 30602, USA

**Keywords:** female preference, mate choice, sexual conflict, direct fitness cost

## Abstract

Sexual conflict occurs when selection to maximize fitness in one sex does so at the expense of the other sex. In the burying beetle *Nicrophorus vespilloides*, repeated mating provides assurance of paternity at a direct cost to female reproductive productivity. To reduce this cost, females could choose males with low repeated mating rates or smaller, servile males. We tested this by offering females a dichotomous choice between males from lines selected for high or low mating rate. Each female was then allocated her preferred or non-preferred male to breed. Females showed no preference for males based on whether they came from lines selected for high or low mating rates. Pairs containing males from high mating rate lines copulated more often than those with low line males but there was a negative relationship between female size and number of times she mated with a non-preferred male. When females bred with their preferred male the number of offspring reared increased with female size but there was no such increase when breeding with non-preferred males. Females thus benefited from being choosy, but this was not directly attributable to avoidance of costly male repeated mating.

## 1. Introduction

Tension between male and female reproductive interests arises when traits that enhance mating success among males impose direct costs on females and/or vice versa [1]. How such costs influence the evolution of mate choice is a key question in evolutionary biology [2] and is likely to depend on the balance of direct costs and indirect benefits. When indirect fitness benefits outweigh direct costs of mating, selection for female preference for successful but harmful males is predicted [3]. However, models show that female choice could also evolve to reduce direct costs of mating (e.g. [4,5]), leading to females that resist mating with ‘costly’ males or that actively choose ‘low-cost’ males.

Sexual conflict has been documented in burying beetles in numerous studies (e.g. [6–9]) and females have good reason to be choosy about male mating partners. Male burying beetles repeatedly mate with females but increased frequency of mating, while improving paternity assurance [10–12], is associated with direct costs to reproductive output [6].

To look at whether females avoid costly males with high repeated mating rates we offered females a dichotomous choice of males on a carcass: one male from a line selected for high repeated mating rates (high line) and one from a line selected for low repeated mating rates (low line). We then mated females to either their preferred or non-preferred partner and measured the number of larvae successfully reared. If avoidance of direct costs of repeated mating is predominantly involved in pre-copulatory female mate choice we predicted that low line males would be preferred over high line males. Alternatively, if indirect benefits of producing high-quality offspring and/or ‘sexy’ sons sired by high line males outweighs associated direct costs, we predicted that high line males would be preferred. We also predicted that mating with high line males would produce fewer offspring whether they were preferred or not.

## 2. Material and methods

### (a) Selection lines

In this experiment, we provided mates from two replicates of lines within which we selected on repeated mating. Full details of the artificial selection protocol can be found in Head *et al.* [6] and details of general beetle husbandry common to all our laboratory beetles in Head *et al.* [13].

We conducted two experiments in series, using beetles from the F_17_ generation of our selection lines. We first tested for effects of selection line from which males were drawn on female mating preference (male mean size does not differ between our lines and did not differ between males in our sample; Welch's two-sample *t*-test, *t*_177_ = 0.294, *p* = 0.769). Virgin females (drawn from a pool of all selection lines and control beetles not under selection) were presented with a simultaneous dichotomous choice between one ‘high line’ male and one ‘low line’ male. Because male size has previously been found to influence mate choice in burying beetles (e.g. [10,14,15]), we matched males within dyads by sorting individuals according to pronotal width (measured to 0.01 mm using vernier calipers). This resulted in a mean pronotal width difference between paired males of 0.17 ± 0.16 mm (mean ± s.d.), with a range of 0.0 to 0.8 mm (mean male size was 5.13 mm).

## 3. Mate choice experiment

Two experimental males were tethered to opposite ends of a plastic container (17 × 12 × 6 cm) with dental floss (tied between the first and second pairs of legs). A defrosted mouse carcass (20–25 g) was placed centrally in the box, randomly oriented (i.e. head or tail), on 2 cm of compost. Tethers permitted males to reach the carcass but not each other. Males were left to acclimate for 30 min before introduction of females. Females had unrestricted access to carcasses and both males. Individual trials lasted for 30 min or until the female made a choice, defined as allowing one male (i.e. the ‘preferred’ male: high line or low line) to mount her. When mounted, the female was removed before intromission and each beetle returned to individual containers.

## 4. Breeding experiment

Twenty-four hours after mate choice trials each female was returned to either her preferred or non-preferred male chosen randomly from each dyad. These pairs were placed in a standard Petri dish lined with filter paper and the number of copulations was recorded for 30 min. Pairs were then transferred to a plastic box (17 × 12 × 6 cm) filled with 3 cm compost and allowed to breed using the same carcass they encountered in the mate choice trial. Larvae resulting from these breeding events were counted and weighed at dispersal to the wandering stage.

## 5. Statistical analysis

We analysed 91 of 113 trials in our dataset in which females were mounted by one of the two available males within 30 min. We used an exact binomial test to test for a female preference based on male line (‘high’ or ‘low’). Then we used a generalized linear model (GLM) with binomial errors to test whether female preference (i.e. did she choose the high line or low line male) was influenced by the size of the female and the size difference between the two males offered (low line male pronotum − high line male pronotum) including replicate as a fixed factor. We tested whether mating frequency was influenced by male preference status (i.e. females mating with preferred or non-preferred male); selection line of the male; female size; male size and all two-way interactions using a GLM with quasi-Poisson errors. Reproductive output (larval number successfully reared) was analysed using a linear model with the same dependent variables as above. All analyses were carried out using R v. 3.1.3 [16].

## 6. Results and discussion

**Figure 1.**
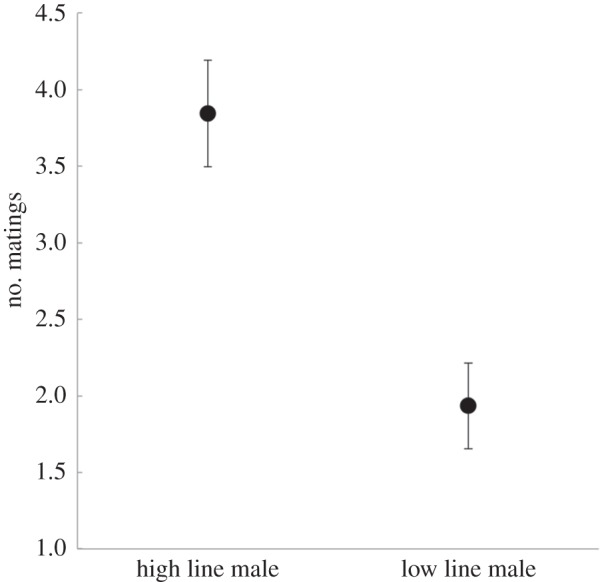
Number of matings (*y*-axis) by selection line of assigned male (*x*-axis). Means and s.e.m.

**Figure 2.**
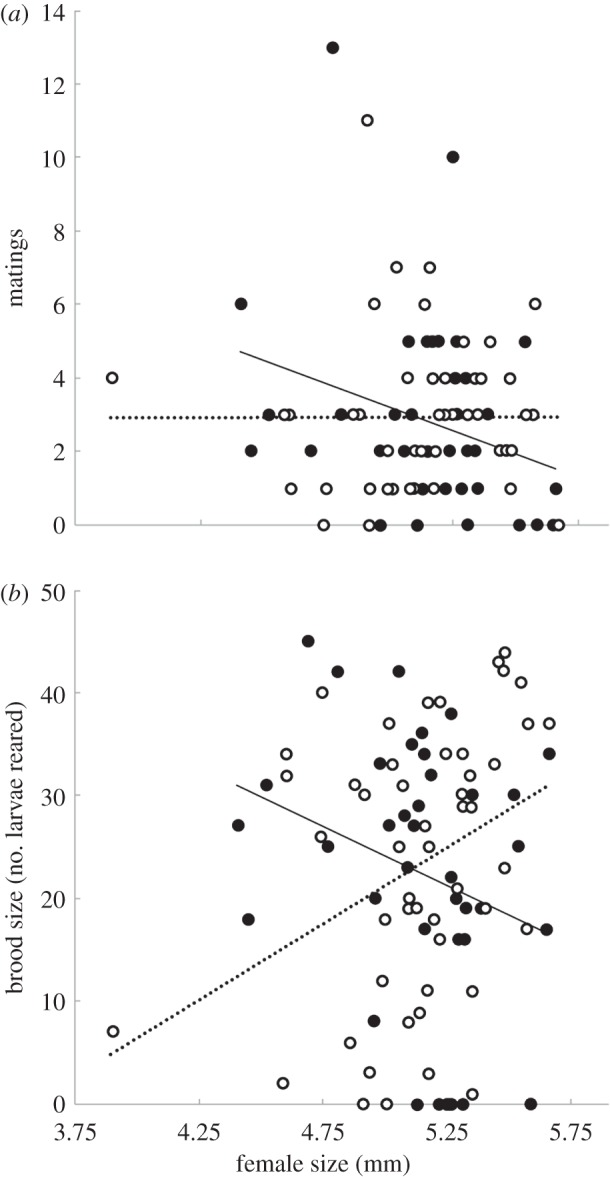
(*a*) The interaction between female size and preference status of her male partner on number of matings. (*b*) The interaction between female size and preference status of her male partner on number of offspring reared (interaction driven by the effect of the preferred male). Open circles and dashed line are preferred males; solid circles and unbroken line are non-preferred males. Lines are inferred from the model fit.

We found no evidence for active pre-copulatory female mate choice based on male selection line (40 ‘high line’ males chosen versus 51 ‘low line’ *p* = 0.295). Nor was there evidence that a female's preference was affected by her size (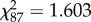, *p* = 0.205); by the size difference between the two males offered (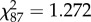, *p* = 0.260); by replicate (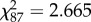, *p* = 0.103), or by any two-way interactions between these variables (all *p*-values > 0.39). Mating frequency was significantly greater in pairs where females were allocated high line compared with low line males (*F*_1,85_ = 21.714, *p* < 0.0001, figure 1), and was positively related to the size of the male (*F*_1,85_ = 4.605, *p* = 0.035). An interaction between female size and male ‘preferred’ status also affected mating frequency: when allocated a non-preferred male larger females mated less often, but when allocated a preferred male there was no relationship between female size and mating rate (*F*_1,85_ = 6.481, *p* = 0.013, figure 2*a*). All other two-way interactions were non-significant (all *p* > 0.2; results of an extended analysis including female origin are presented in the electronic supplementary material, table S1).

Reproductive output was also subject to the interaction between female size and male preferred status: there was a positive relationship between offspring number reared and female size but only in breeding pairs involving a preferred male (linear model (LM), *F*_1,87_ = 8.569, *p* < 0.004, figure 2*b*). None of the other main effects (i.e. male size and selection line) or interactions was significant (all *p* > 0.12). We found no direct relationship between mating frequency and number of offspring reared (LM, *F*_1,89_ = 0.403, *p* = 0.688).

One explanation for the patterns of mating and reproductive output is that resistance to mating interferes with effective parenting. Male attempts to coerce females into extra matings might be more effectively resisted by a larger female. However, the relative pay-off of mating with preferred males might not be higher for larger females if smaller females are less fecund. In the closely related burying beetle, *Nicrophorus quadripunctatus*, Suzuki [15] found that females of intermediate size repelled a greater proportion of smaller males' mating attempts than those of larger males. This pattern could occur if females resist repeated mating according their own coercive ability. There is also evidence in *N. vespilloides* for female coercion directed at preventing males calling extra-pair females to large carcasses [17] and females do actively resist matings by kicking at males and attempting to prevent them from engaging genitalia.

Females appear to be responding to a male attribute because the effect of female size on mating frequency is confined to non-preferred males. One possibility is cues indicating reproductive readiness or parental prowess. In nature, female burying beetles have opportunities to choose among males in two contexts: choosing among males releasing pheromones away from a carcass [18], and/or among males that are present on a carcass suitable for breeding. Burying beetle females can detect and respond to differences among males in the expression of cuticular chemicals [19] and in our study differences in male suitability to parent might have been discernable by females. However, in the context of male–male competition for matings on a carcass in nature, it is possible that females have little opportunity to exercise mate choice *per se*.

When males compete on a carcass, females suffer increased matings driven by paternity protection behaviour [10] and this might have provided the source of selection for active mate choice to evolve in responding, or not responding, to calling males [20]. In this context, females could mate with a preferred male (calling without a carcass) and avoid direct costs of repeated mating entirely when they subsequently find a carcass and breed alone.

## Data accessibility

Data are deposited at Dryad: http://dx.doi.org/10.5061/dryad.k765g.
